# P-1271. Emergence of Daptomycin Heteroresistance of in Methicillin- resistant Staphylococcus aureus

**DOI:** 10.1093/ofid/ofaf695.1461

**Published:** 2026-01-11

**Authors:** Jagjeet Kaur, Maryssa Trupiano, Britney Aldrich, Geehan Suleyman

**Affiliations:** Henry Ford Health, Detroit, MI; henry Ford Health System, Detroit, Michigan; henry Ford Health System, Detroit, Michigan; Henry Ford Health, Detroit, MI

## Abstract

**Background:**

Heteroresistance, characterized by resistant subpopulations within a predominantly antimicrobial-susceptible bacterial population, can arise under antibiotic selection pressure. However, its role in the development of daptomycin (DAP) resistance in S. aureus (SA) and its impact on clinical outcomes remain poorly defined. Furthermore, standardized methods for detecting DAP heteroresistance are lacking. The aim of this study was to assess the prevalence of DAP heteroresistance among DAP-susceptible (DS) SA strains that subsequently developed DAP-nonsusceptibility (DNS).

**Methods:**

In vitro susceptibility testing for DAP was performed by the clinical microbiology lab using standardized methods. Modified population analysis profiles (PAPs) were conducted to assess heteroresistance using brain heart infusion (BHI) agar plates supplemented with varying concentrations of DAP ranging from 0 to 4 μg/mL. 100 μL of 105 and 106 CFU/mL suspension of each strain was spiral plated (Eddy Jet 2W spiral plater; IUL micro) onto DAP-containing BHI agar plates and incubated at 37°C for 48 hrs. The colonies were counted after 48 hrs using the Sphere Flash colony counter (IUL micro). PAPs were generated by plotting the log10 CFU/ml against the antibiotic concentrations. A comparison of the area-under-the-curve (AUC) of the control strain (ATCC 29213) was performed with the DS isolates by visual inspection using GraphPad software.

**Figure:**
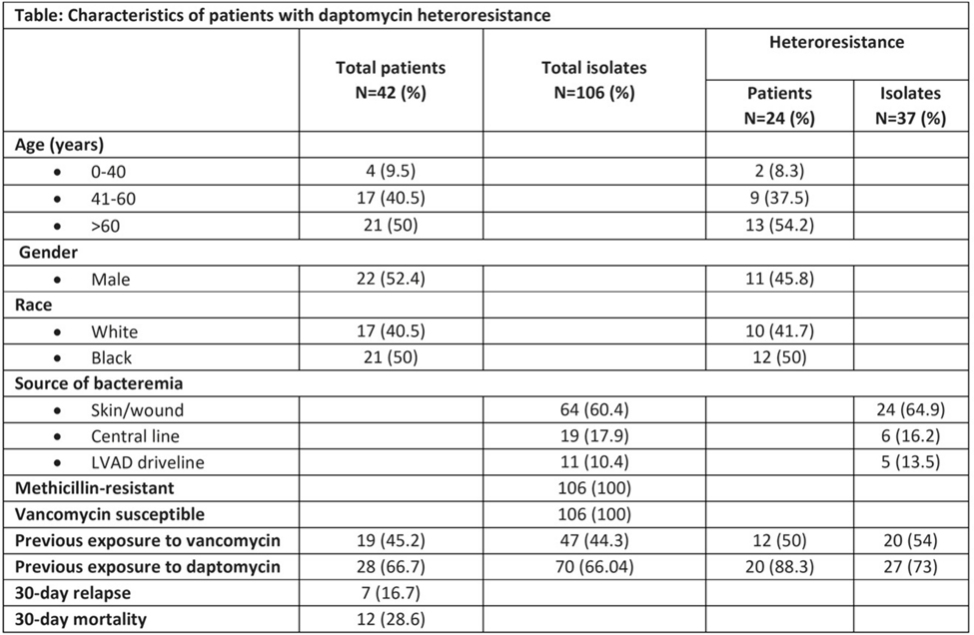

**Results:**

106 DS SA strains from 42 patients were analyzed (Table). Most patients were Black males with age >60 years. The most common source of bacteremia was the skin (60%). All strains were methicillin-resistant (MRSA) and vancomycin (VAN) susceptible. Among the 106 isolates, heteroresistance was identified in 37 (35%) strains from 24 (57%) patients. Of the 37 heteroresistant isolates, 27 (73%) were exposed to DAP and 20 (54%) to VAN, while 10 (27%) had no antibiotic exposure. 30-day relapse was common and 30-day mortality was high.

**Conclusion:**

n this large cohort of patients with DS SA who subsequently developed DNS, heteroresistance was observed in over a third of isolates. While the majority had prior exposure to DAP, a quarter did not. Further research is necessary to elucidate the underlying mechanisms of heteroresistance and its impact on clinical outcomes.

**Disclosures:**

All Authors: No reported disclosures

